# miRNA profiling in intrauterine exosomes of pregnant cattle on day 7

**DOI:** 10.3389/fvets.2022.1078394

**Published:** 2022-12-20

**Authors:** Yaying Zhai, Qiaoting Shi, Qiuxia Chu, Fuying Chen, Yajie Feng, Zijing Zhang, Xinglei Qi, Danny Arends, Gudrun A. Brockmann, Eryao Wang, Shijie Lyu

**Affiliations:** ^1^Institute of Animal Husbandry and Veterinary Science, Henan Academy of Agricultural Sciences, Zhengzhou, Henan, China; ^2^College of Animal Science and Veterinary Medicine, Henan Agricultural University, Zhengzhou, China; ^3^Center of Animal Husbandry Technical Service in Biyang, Zhumadian, China; ^4^Department of Applied Sciences, Northumbria University, Newcastle upon Tyne, United Kingdom; ^5^Albrecht Daniel Thaer-Institute of Agricultural and Horticultural Sciences, Humboldt-Universität zu Berlin, Berlin, Germany; ^6^The Shennong Laboratory, Zhengzhou, Henan, China

**Keywords:** bovine, uterine flushing, receptive endometrium, apoptosis, proliferation

## Abstract

Intrauterine exosomes have been identified to be involved in the embryo development and implantation. The aim of this study was to explore the role of miRNAs in intrauterine exosomes in bovine pregnancy. Intrauterine exosomes were collected from uterine flushing fluids of three donor and three recipient Xianan cows 7 days after fertilization. Intrauterine exosomes miRNAs were extracted and the exosomal miRNAs expression levels were analyzed. Sixty miRNAs differed significantly in their amounts between donors and recipients (*p-value* < 0.05, |log2(FoldChange)| > 1). Twenty-two miRNAs were upregulated and 38 downregulated in the group of donor cows. The bta-miR-184 was the most significant (*P*_Benjamini-Hochberg_ < 0.001). A total of 9,775 target genes were predicted using the 60 miRNAs. GO and KEGG analysis showed that the target genes were enriched in several biological processes or pathways associated with embryo implantation and endometrial development, such as cell adhesion, cell junction, focal adhesion, and Rap1 signaling pathway. Our findings suggest that, in cattle early pregnancy stage, these differently expressed miRNAs in intrauterine exosomes involved in embryo implantation and endometrial development, which may exert a significant effect and influence the uterine microenvironment for embryo implantation. These results could provide reference for screening and exploring the intrauterine exosomal miRNA affecting embryo implantation.

## Introduction

Low conception rate is a problem which significantly reduces the economic benefits of cattle industry. It is reported that more than 50% embryo loss occurs during the first week of pregnancy (1). The establishment of pregnancy requires not only the normal development of the embryo itself but also a favorable environment for embryo implantation (2). During the implantation stage of bovine embryos, uterine fluid supports embryonic development and survival (3). To enlighten potential causes for bovine low conception rate, it is necessary to better understand the interaction between the early embryo and the uterine fluid.

Exosomes are 50–150 nm extracellular vesicles secreted by various cell types. They are a media for intercellular communication (4, 5). They contain nucleic acids, proteins, and lipids, and play an important role in adhesion, proliferation, apoptosis, inflammation, and immune response in recipient cells (6, 7). Growing evidence has shown that exosomes are important for the establishment of pregnancy *via* affecting oogenesis, oocyte maturation and fertilization, embryo-maternal cross talk, and embryo implantation (7, 8).

In uterine flushing fluids (UFs), exosomes have been identified and found to be involved in the embryo development and implantation as well (9, 10). In sheep, endometrial exosomes regulate the secretion of interferon tau (IFNT), a pregnancy recognition signal in ruminants (11). In cattle, treatment of endometrial epithelial cells with exosomes isolated from the UFs of pregnant cattle at day 17 increased the expression of apoptosis-related genes and IFNT-stimulated genes (10, 12). Exosomes are particularly enriched in miRNAs, which can regulate the translation of mRNA to protein in the recipient cells. Thereby, exosomes with their content are most likely factors influencing the establishment of pregnancy (13). For example, in humans, miR-30d from exosomes in the uterine lumen is increased during the window of implantation. Mouse *in vitro* experiments showed that exosome-associated miR-30d was taken up by the trophectoderm of blastocysts and which contributed to increase trophectoderm adhesion (14). In goats, exosomal miRNAs in UFs were found to be required for uterine receptivity and embryo implantation (15).

After fertilization, the bovine morula enters the uterus on days 4–5 where it forms a blastocyst by day 7. We hypothesize that miRNAs of exosomes in the intrauterine fluid between day 5 and 7 after fertilization might be required for the establishment of a favorable uterus environment for the embryo implantation on day 7. Therefore, we aim to explore potential functions of miRNAs in intrauterine exosomes for the implantation of bovine embryos. In this study, the diversity and amounts of miRNAs in the exosomes of intrauterine fluids of donor and recipient cows on day 7 after fertilization were investigated. The result of this study could provide reference for screening and exploring the intrauterine exosomal miRNA affecting embryo implantation, and provide the basis for further clarification of intrauterine fluids exosomes in the early pregnancy regulation of cattle.

## 2. Materials and methods

### 2.1. Collection of bovines flushed uterine fluids

All animal procedures in this study were performed in accordance with the guidelines of the Committee for Experimental Animals at Henan Academy of Agricultural Sciences. Ten Xianan cows (a beef cattle breed in China) were selected as donors for synchronous estrus and superovulation. The hormones used for superovulation are follicle-stimulating hormone (FSH) and prostaglandin (PG), which are administered by intramuscular injection. FSH is administered over 4 consecutive days, twice daily in decreasing doses. Over the 4 days, FSH is administered every 12 h, at 7 am and 7 pm. The treatment protocol is as follows: day 1, 70 mg every 12 h; day 2, 50 mg every 12 h; day 3, 30 mg every 12 h; day 4, 20 mg every 12 h. At 7 pm on day 3 of FSH treatment, 0.1 mg PG was injected and repeated 12 h later. Estrus can be expected 36–48 h later. The artificial insemination was performed after superovulation. Another 23 Xianan cows were selected as recipients for synchronous estrus. On the 7th day after artificial insemination of donors, 100 ml bovine UFs were collected by uterine flushing using sterile Gibco Minimum Essential Media with 0.7% 200 mM Tris buffer (pH = 7.2). Three donor cows with well-developed blastocysts were used for further analysis (Table 1). Three recipient cows having similar body weight and age were selected as matched controls. All UF samples were stored at −80°C until further analysis.

**Table 1:** The information of the donor and recipient cows.

**Individual**	**Age (year)**	**Body weight (kg)**	**Number of blastocysts obtained**
Donor cow No.1	4	602	4
Donor cow No.2	3	486	2
Donor cow No.3	6	658	4
Recipient cow No. 1	4	530	–
Recipient cow No. 2	4	482	–
Recipient cow No. 3	6	610	–

### 2.2. Isolation of exosomes from uterine flushing fluids

Each UF sample was thawn at 37°C and centrifuged at 2,000 g for 30 min at 4°C. The supernatant was collected and then centrifuged at 12,000 g for 45 min at 4°C. After filtering through a 0.45 μm filter (Millipore, USA), the resulting supernatant was centrifuged at 110,000 g for 70 min at 4°C (Hitachi CP100MX, Japan). The pellet was suspended in 10 ml pre-cooling PBS and centrifuged at 110,000 g for 70 min at 4°C again. Finally, the pellet was resuspended in pre-cooling 50 μL PBS and stored at −80°C until use.

### 2.3. Nanoparticle tracking analysis and transmission electron microscopy

Nanoparticle tracking analysis (NTA) was performed for all samples by ZetaView S/N 17-310 (Particle metrix, Germany). One sample was randomly selected to detect the morphology using transmission electron microscopy (TEM). For this sample, a 10 μL aliquot was placed on a carbon-coated copper grid for 1 min and stained with a drop of 2% phosphotungstic acid for 1 min. After blotting off the excess liquid using filter paper, the grids were dried for 15 min at room temperature. Micrographs were then observed using a transmission electron microscope (Hitachi HT-7700, Japan).

### 2.4. Small RNA library construction

Total RNA in exosomes from uterine flushing fluids was extracted using the miRNeasy Serum/Plasma Kit (Qiagen, Germany) according to the manufacturer's instructions. The integrity and concentration were determined by the Agilent 2100 Bioanalyzer (Agilent Technology, USA). A total of 10 ng total RNA was used to construct the small RNA library using the TruSeq Small RNA Sample Prep Kits (Illumina, USA). In brief, adapters were ligated to both ends of the total RNA. Complementary DNA (cDNA) was then synthesized through reverse transcribe. Pieces between 147 and 157 bp were collected to construct a small RNA library. Agilent Technologies 2100 Bioanalyze was used to check the size and purity of the library. Finally, the library was sorted using the Illumina HiSeq X Ten platform. In the end, 150 bp paired-end reads were generated. Small RNA sequencing and analysis were performed by OE biotech (Shanghai, China). Data are available from the GEO database GSE216746.

### 2.5. Bioinformatics analysis

Clean reads were obtained by removing reads with low quality, 5' primer contaminants and poly (A). Reads without 3' adapter, insert tag, shorter than 15 nt and longer than 41 nt were also filtered. Q20 quality control was performed on the sequence to remove low quality reads where Q20 did not reach 80%. The length distribution of the clean reads in the reference genome (ARS-UCD 1.2) was determined. Non-coding RNAs consisting of rRNAs, tRNAs, small nuclear RNAs, and small nucleolar RNAs were aligned and then subjected to the BLAST search against Rfam v.10.1 (16). The known miRNAs were annotated by aligning against the miRBase v.21 database (17). Unannotated small RNAs were analyzed by miRdeep2 to predict novel miRNAs (18).

MiRNA expression levels were estimated by TPM (transcript per million). Differential expression analysis between two groups was conducted using the “DESeq” package in R (19). *P*-values were corrected for multiple testing using the Benjamini-Hochberg method (P_BH_). The miRNAs with *p*-value < 0.05 and |log_2_FoldChange|>1 were considered as significantly differentially expressed miRNAs. Prediction of the target genes for the identified differentially expressed miRNAs was performed using the miRanda program (20) with the parameter of S ≥ 150, ΔG ≤ −30 kcal/mol and demand strict 5' seed pairing. Gene Ontology (GO) enrichment and Kyoto Encyclopedia of Genes and Genomes (KEGG) pathway enrichment analysis of these predicted target genes were performed using R based on the hypergeometric distribution (21). The false discovery rate of 0.05 and ListHits of five were set as the selection threshold for GO and KEGG analyses.

## 3. Results

### 3.1. Exosome isolation and characterization

**Figure 1:**
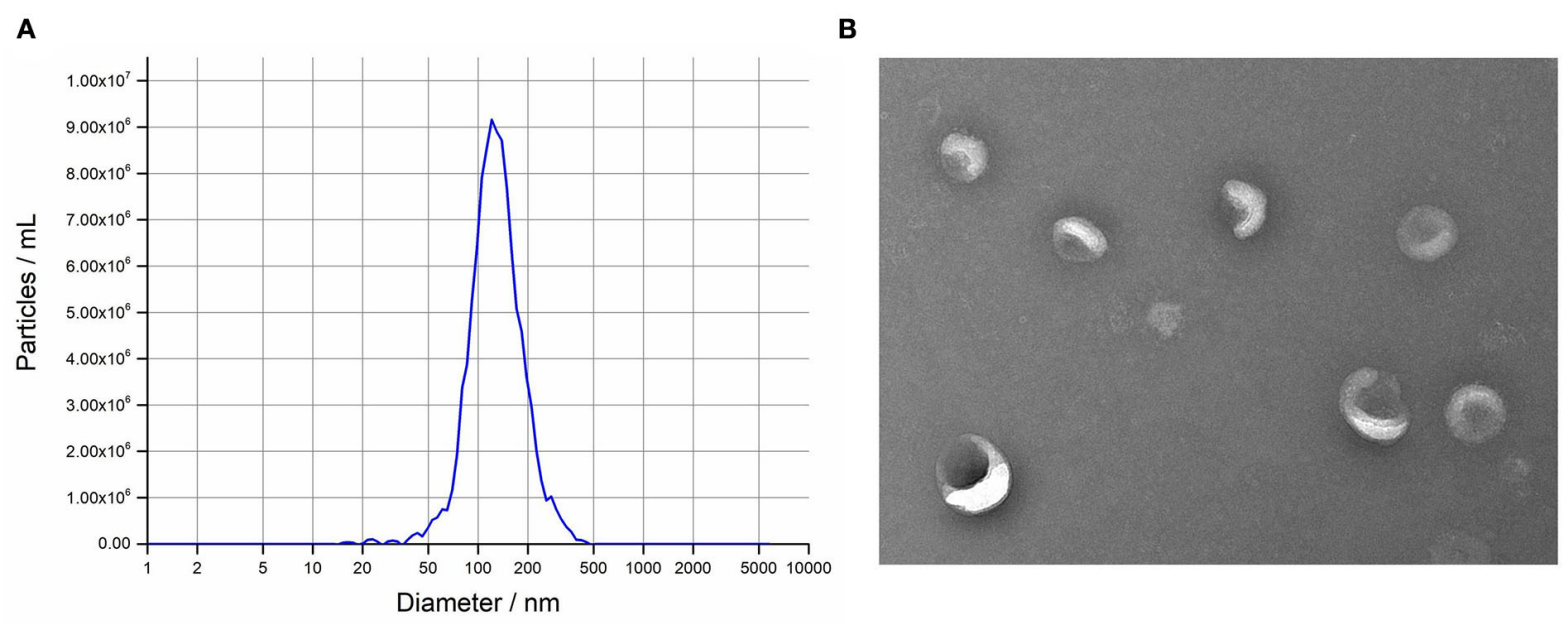
Isolation and characterization of intrauterine exosomes. **(A)** Transmission electron microscopy images showed that the isolated vesicles were oval or bowl-shaped. Scale bar, 200 nm. **(B)** Nanoparticle tracking analysis results demonstrated that the particle size distribution was in exosome-enriched fractions.

The isolated exosomes from uterine flushing fluids were identified by NTA and TEM. The isolated vesicles have a classic exosome size. On average, the most widely distributed particle diameter of the six samples was 126.9 nm (Figure 1A). The result of TEM showed that the vesicles were bowl- shaped, which have the classic exosome morphology (Figure 1B).

### 3.2. Differential miRNA expression analysis

**Figure 2:**
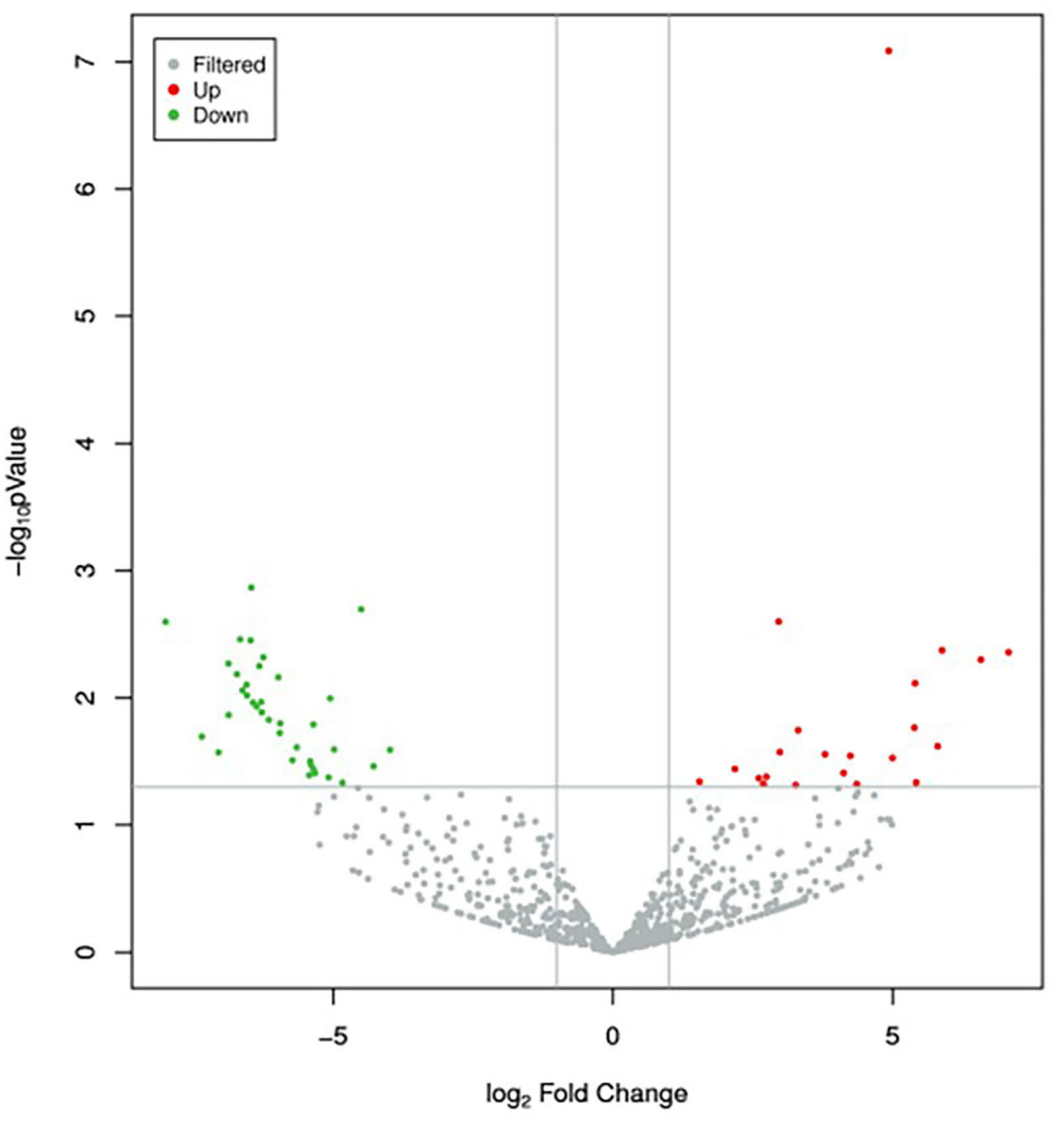
Volcano plot of differentially expressed miRNAs between the two groups. Red dots and green dots denote miRNAs were up-regulated and down-regulated in the group of donor cows, respectively. The significance cut-off was set to a *p*-value of 0.05 and |log_2_FoldChange|of 1.

A total of 895 unique miRNAs were differentially expressed between the donor and recipient cows. Among them, 60 miRNAs were significantly differentially expressed (*p-*value<0.05, |log_2_FoldChange|>1) (Figure 2). Twenty-two miRNAs were upregulated and 38 miRNAs downregulated in the group of donor cows (Table 2). The top 10 differentially expressed miRNAs were novel352_mature, bta-miR-1388-3p, bta-miR-211, novel548_mature, novel530_mature, bta-miR-1224, novel275_star, novel190_mature, novel170_mature, bta-miR-2904.

**Table 2:** Significantly differentially expressed miRNAs in intrauterine exosomes between the donor and recipient cows on 7 days after fertilization (donor group vs. recipient group).

**miRNA_ID**	**|log2 FoldChang|**	***P*-value**	**P_BH_**	**Regulation**
bta-miR-184	4.931	<0.001	<0.001	Up
bta-miR-660	2.964	0.003	0.382	Up
bta-miR-211	7.074	0.004	0.382	Up
bta-miR-2285bc	5.885	0.004	0.382	Up
bta-miR-2904	6.575	0.005	0.382	Up
novel399_mature	5.402	0.008	0.408	Up
novel490_mature	5.387	0.017	0.503	Up
bta-miR-129-5p	3.313	0.018	0.510	Up
novel527_mature	5.805	0.024	0.606	Up
bta-miR-10225b	2.987	0.027	0.606	Up
bta-miR-154c	3.791	0.028	0.610	Up
novel459_mature	4.244	0.029	0.614	Up
bta-miR-191b	4.998	0.030	0.621	Up
bta-miR-504	2.179	0.036	0.664	Up
bta-miR-365-5p	4.124	0.039	0.686	Up
bta-miR-2387	2.746	0.042	0.698	Up
novel288_mature	2.611	0.043	0.698	Up
bta-miR-2285q	5.418	0.046	0.711	Up
bta-miR-200b	1.549	0.046	0.711	Up
novel30_mature	4.359	0.048	0.711	Up
bta-miR-1306	3.269	0.048	0.711	Up
bta-miR-885	2.692	0.048	0.711	Up
novel439_mature	6.457	0.001	0.382	Down
novel142_mature	4.494	0.002	0.382	Down
novel352_mature	7.989	0.003	0.382	Down
novel190_mature	6.66	0.003	0.382	Down
novel145_mature	6.472	0.004	0.382	Down
novel530_mature	6.866	0.005	0.382	Down
novel159_mature	6.245	0.005	0.382	Down
novel504_mature	6.315	0.006	0.382	Down
novel275_star	6.714	0.007	0.405	Down
novel425_mature	5.975	0.007	0.405	Down
novel165_mature	6.545	0.008	0.408	Down
novel170_mature	6.614	0.009	0.427	Down
novel138_mature	6.536	0.010	0.430	Down
novel484_mature	5.049	0.010	0.430	Down
novel473_mature	6.427	0.011	0.430	Down
novel471_mature	6.283	0.011	0.430	Down
novel71_mature	6.365	0.012	0.430	Down
novel155_mature	6.361	0.012	0.430	Down
novel406_mature	6.272	0.013	0.457	Down
bta-miR-1224	6.863	0.014	0.462	Down
novel513_mature	6.144	0.015	0.485	Down
novel180_mature	5.941	0.016	0.491	Down
novel317_mature	5.35	0.016	0.491	Down
novel378_mature	5.949	0.019	0.519	Down
bta-miR-1388-3p	7.343	0.020	0.538	Down
novel577_mature	5.645	0.025	0.606	Down
novel318_mature	4.978	0.025	0.606	Down
novel311_mature	3.978	0.026	0.606	Down
novel548_mature	7.047	0.027	0.606	Down
novel150_mature	5.723	0.031	0.630	Down
novel259_mature	5.408	0.032	0.630	Down
novel408_mature	5.391	0.034	0.660	Down
novel186_mature	4.273	0.034	0.660	Down
novel110_mature	5.351	0.036	0.664	Down
novel526_mature	5.321	0.039	0.686	Down
novel119_mature	5.423	0.040	0.698	Down
bta-miR-1343-5p	5.077	0.042	0.698	Down
novel405_mature	4.828	0.046	0.711	Down

### 3.3. GO enrichment and KEGG pathway analyses of predicted target genes of the differentially expressed miRNAs

Target genes of the differentially expressed miRNAs were predicted using the miRanda program. Subsequently, GO enrichment analysis was performed to explore the potential functions of these target genes, which was used to infer the function of the differentially expressed miRNAs. The GO analysis results were assigned to biological processes (BP), cellular component (CC), and molecular function (MF).

**Figure 3:**
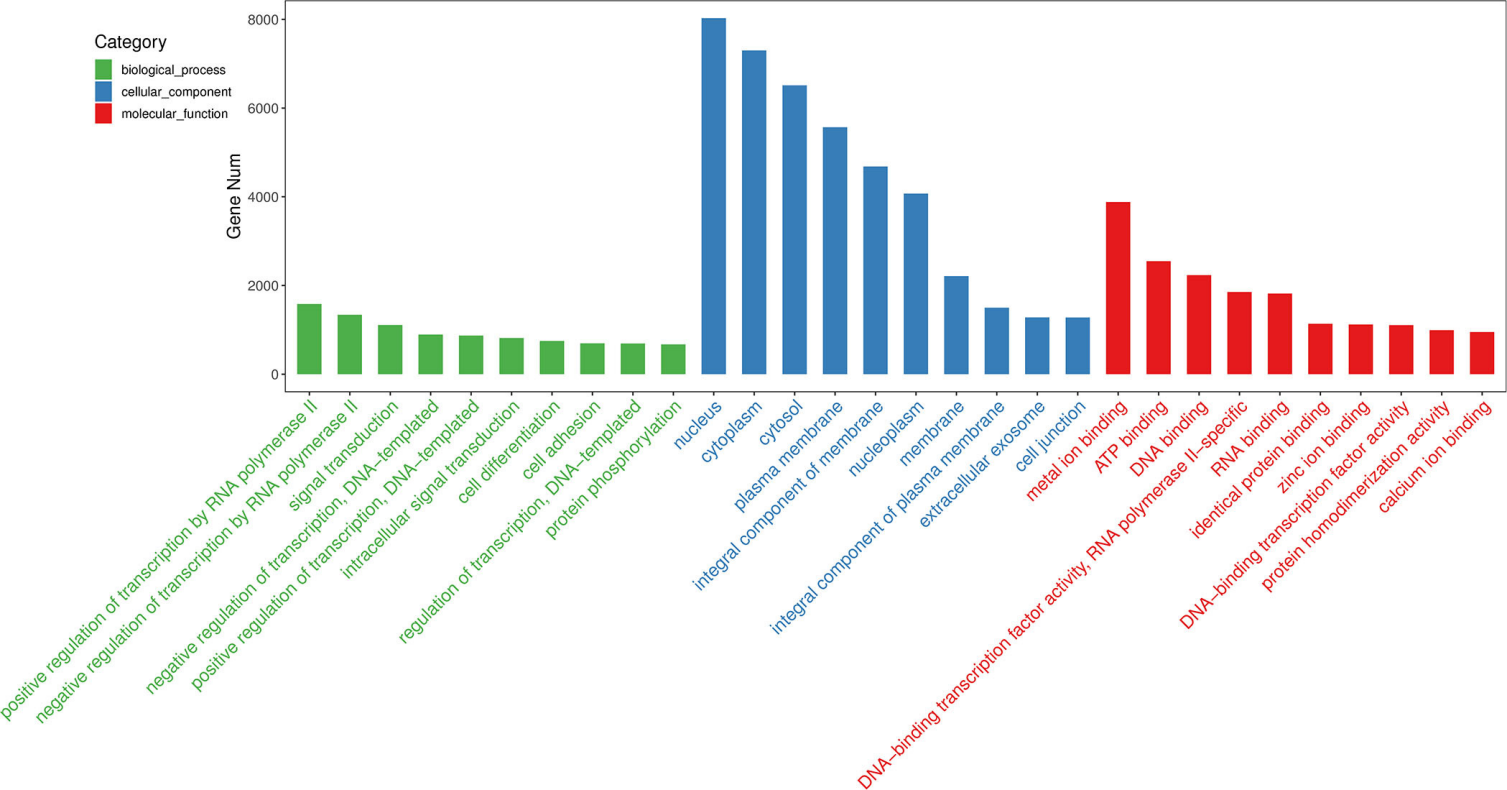
GO analysis of predicted target genes of differentially expressed miRNAs. Top 10 terms of biological process, cellular component, and molecular function were shown.

**Figure 4:**
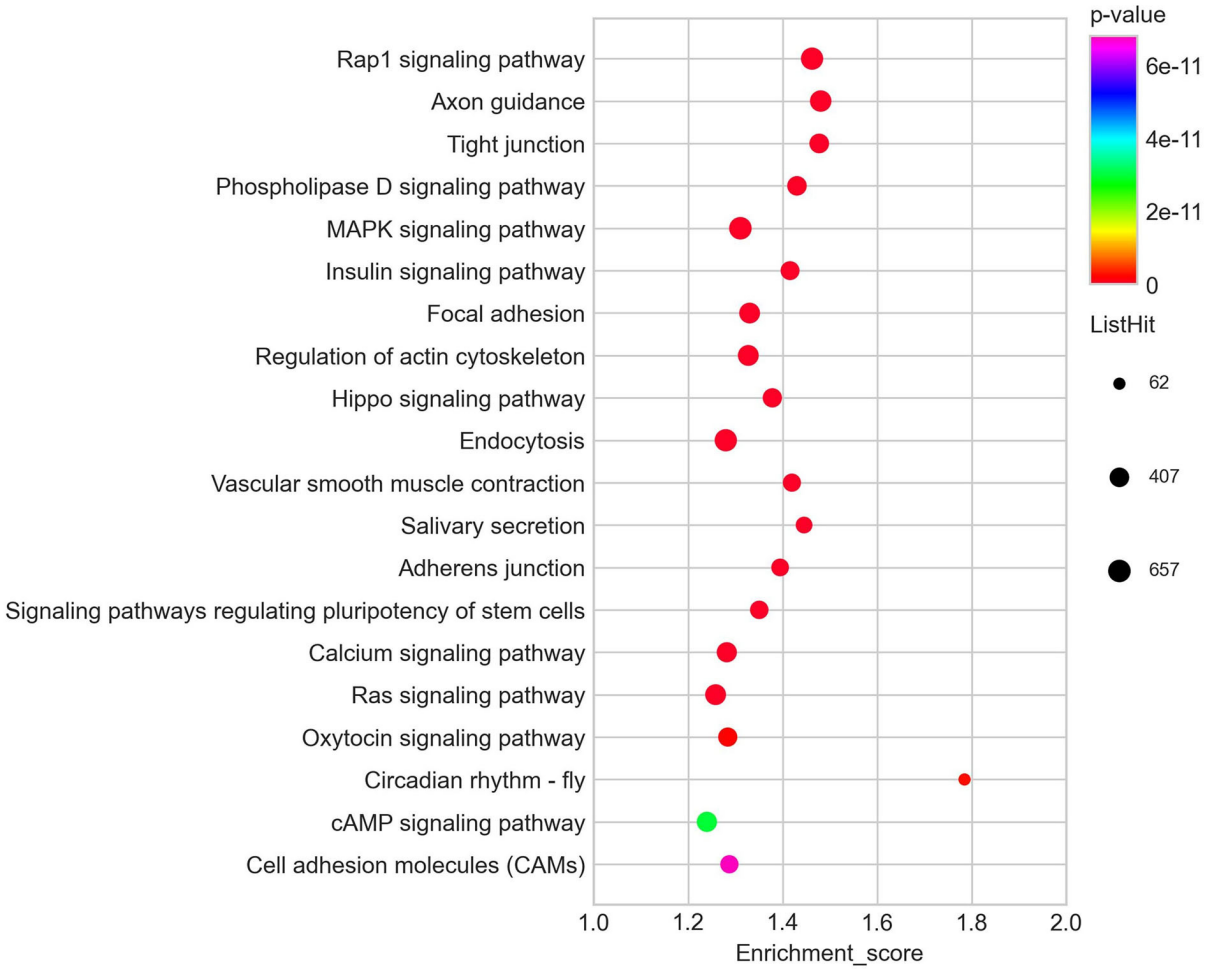
Top 20 KEGG pathways of predicted target genes of differentially expressed miRNAs.

Among the 60 differentially expressed miRNAs, 55 miRNAs were available to predict 9775 non-repeating target genes. 1568, 299, and 476 GO terms that were significant for BP, CC, and MF, respectively. Based on the top 30 GO terms (Figure 3), the GO analysis showed that the target genes were enriched in cell differentiation, cell adhesion, and cell junction. These processes play an important role in embryo implantation (22). For KEGG analysis, eighty-three terms were significant. Some KEGG pathways in the top 20 terms, such as focal adhesion, adherens junction, tight junction, Rap1 signaling pathway and MAPK signaling pathway, were related to embryo implantation and endometrial development (23–27) (Figure 4).

## 4. Discussion

In the current study, miRNA expression profiles in intrauterine exosomes of donor and recipient cows on day 7 after fertilization were characterized. A total of 60 differentially expressed miRNAs were found. GO and KEGG analysis showed that the target genes of these miRNAs were mainly enriched in several biological processes or pathways associated with embryo implantation and endometrial development. These results demonstrated that the miRNAs in intrauterine exosomes could be significantly changed by the embryos on day 7 and might participate in the embryo implantation.

Embryo implantation is a complex process. The miRNA-mRNA regulatory system has been found to play an important role in the successful implantation of embryos (28). In this study, GO analysis showed that the top 30 biological processes were mainly enriched in cell differentiation, cell adhesion and cell junction, which are related to embryo implantation (29–32). For the KEGG analysis, the most significant term is Rap1 signaling pathway. The Rap1 signaling pathway has many important biological functions such as control of cell adhesion, cell junction, and cell proliferation (23, 33, 34). In addition, among the top 20 enriched pathways, some pathways such as MAPK signaling pathway, focal adhesion, adherens junction, and cell adhesion molecules provided more evidence that the differentially expressed miRNAs identified in this study were involved in the embryo implantation and endometrial development.

It has been reported that some miRNAs in the embryo- and endometrium-derived exosomes affect embryo implantation (15, 35, 36). A recent study, which has a similar research strategy as our study, found nine miRNAs in the UFs were differently expressed between inseminated and non-inseminated Japanese Black cows (37). Nevertheless, no overlapping miRNAs were found between this study and ours. This consequence might be due to the difference in the cattle breeds.

This study demonstrated that the presence of intrauterine exosomes, especially the miRNAs in intrauterine exosomes, played an important role in the early pregnancy in cattle. But the origin of exosomes was not determined. Due to the limitation of sample sources, the sample size of this experiment is small. In order to reduce the experimental error caused by large individual differences, we chose cows with similar weight and age as much as possible. In order to avoid the occurrence of false positives, we used the analysis method of multiple comparisons to analyze the experimental data. In spite of we selected donors and recipients with similar body weight and age, but the influence of individual difference on the results cannot be ruled out, as well as a possible effect stemming from the low sample size used in this study. These issues lead to only one miRNA (bta-miR-184) being significantly differential expressed after multiple testing correction. The deletion of miR-184 in the process of egg cell expulsion and early embryonic development in drosophila melanogaster can lead to a variety of serious pathologies, which can lead to the loss of egg function. Targeted regulation of these genes by miR-184 may be necessary for oogenesis and early embryo development (38). MiR-184 has been reported to play roles in embryo implantation. In dairy goats, high throughput sequencing results of miRNA in the endometrial receptivity (RE) receptivity (PE) of dairy goats showed that the enrichment of miR-184 and RE was 31 times higher than that of PE (*P* < 0.01) (39), and miR-184 promotes the apoptosis of endometrial epithelial cells by downregulating STC2 *via* the RAS/RAF/MEK/ERK pathway and is involved in the establishment of receptive endometrium (40).

For the differentially expressed miRNAs (*p-*value < 0.05), although the function of them was not verified, the GO and KEGG analysis indicated that they might be involved in the process of embryo implantation in cattle. The impact of these miRNAs on the establishment of pregnancy in cattle warrants further investigations.

## 5. Conclusion

In conclusion, 60 miRNAs showing significant differences in expression were detected by comparing intrauterine exosomes of donor vs. recipient cows on 7 days after fertilization. GO functional enrichment analysis and KEGG signaling pathway analysis showed that these miRNAs were involved in biological processes related to cell adhesion and focal adhesion. This strongly suggests that miRNAs in intrauterine exosomes exert some kind of influence on the early stages of cow pregnancy. Our study provides an experimental basis to screen and explore the intrauterine exosomal miRNA affecting embryo implantation.

## Data availability statement

The datasets presented in this study can be found in online repositories. The name of the repository and accession number can be found at: GEO, NCBI; GSE216746.

## Ethics statement

The animal study was reviewed and approved by Committee for Experimental Animals at Henan Academy of Agricultural Sciences.

## Author contributions

YZ: methodology, investigation, data curation, and writing–original draft. QS and FC: methodology, data curation, and formal analysis. QC: methodology, investigation, and data curation. YF, ZZ, and XQ: methodology and data curation. DA and GB: formal analysis and writing–review and editing. EW: conceptualization, funding acquisition, resources, supervision, and writing–review and editing. SL: conceptualization, methodology, investigation, data curation, formal analysis, writing–original draft, and writing–review and editing. All authors contributed to the article and approved the submitted version.

## References

[1] Wiltbank MC Baez GM Garcia-Guerra A Toledo MZ Monteiro PL Melo LF. Pivotal periods for pregnancy loss during the first trimester of gestation in lactating dairy cows. Theriogenology. (2016) 86:239–53. 10.1016/j.theriogenology.2016.04.037 27238438

[2] Liao XG Li YL Gao RF Geng YQ Chen XM Liu XQ. Folate deficiency decreases apoptosis of endometrium decidual cells in pregnant mice *via* the mitochondrial pathway. Nutrients. (2015) 7:1916–32. 10.3390/nu7031916 25781218 PMC4377890

[3] Silva F da Silva GF Vieira BS Neto AL Rocha CC Lo Turco EG. Peri-estrus ovarian, uterine, and hormonal variables determine the uterine luminal fluid metabolome in beef heifers. Biol Reprod. (2021) 105:1140–53. 10.1093/biolre/ioab149 34350935

[4] Kim GB Shon O-J Seo M-S Choi Y Park WT Lee GW. Mesenchymal stem cell-derived exosomes and their therapeutic potential for osteoarthritis. Biology. (2021) 10:285. 10.3390/biology10040285 33915850 PMC8066608

[5] Thery C. Exosomes: secreted vesicles and intercellular communications. Biol Rep. (2011) 3:15. 10.3410/B3-15 21876726 PMC3155154

[6] Kalluri R LeBleu VS. The biology, function, and biomedical applications of exosomes. Science. (2020) 367:105247. 10.1126/science.aau6977 32029601 PMC7717626

[7] O'Neil EV Burns GW Spencer TE. Extracellular vesicles: novel regulators of conceptus-uterine interactions? Theriogenology. (2020) 150:106–12. 10.1016/j.theriogenology.2020.01.083 32164992 PMC8559595

[8] Capra E Lange-Consiglio A. The biological function of extracellular vesicles during fertilization, early embryo-maternal crosstalk and their involvement in reproduction: review and overview. Biomolecules. (2020) 10:1510. 10.3390/biom10111510 33158009 PMC7693816

[9] Burns G Brooks K Wildung M Navakanitworakul R Christenson LK Spencer TE. Extracellular vesicles in luminal fluid of the ovine uterus. PLoS ONE. (2014) 9:e90913. 10.1371/journal.pone.0090913 24614226 PMC3948691

[10] Kusama K Nakamura K Bai R Nagaoka K Sakurai T Imakawa K. Intrauterine exosomes are required for bovine conceptus implantation. Biochem Biophys Res Commun. (2018) 495:1370–5. 10.1016/j.bbrc.2017.11.176 29196267

[11] Ruiz-Gonzalez I Xu J Wang X Burghardt RC Dunlap KA Bazer FW. Exosomes, endogenous retroviruses and toll-like receptors: pregnancy recognition in ewes. Reproduction. (2015) 149:281–91. 10.1530/REP-14-0538 25526899

[12] Nakamura K Kusama K Bai R Sakurai T Isuzugawa K Godkin JD. Induction of ifnt-stimulated genes by conceptus-derived exosomes during the attachment period. PLoS ONE. (2016) 11:e0158278. 10.1371/journal.pone.0158278 27351483 PMC4924817

[13] Czernek L Düchler M. Exosomes as messengers between mother and fetus in pregnancy. Int J Mol Sci. (2020) 21:4264. 10.3390/ijms21124264 32549407 PMC7352303

[14] Vilella F Moreno-Moya JM Balaguer N Grasso A Herrero M Martínez S. Hsa-Mir-30d, secreted by the human endometrium, is taken up by the pre-implantation embryo and might modify its transcriptome. Development. (2015) 142:3210–21. 10.1242/dev.124289 26395145

[15] Xie Y Liu G Zang X Hu Q Zhou C Li Y. Differential expression pattern of goat uterine fluids extracellular vesicles mirnas during peri-implantation. Cells. (2021) 10:2308. 10.3390/cells10092308 34571957 PMC8470123

[16] Griffiths-Jones S Bateman A Marshall M Khanna A Eddy SR. Rfam: an RNA family database. Nucleic Acids Res. (2003) 31:439–41. 10.1093/nar/gkg0006 12520045 PMC165453

[17] Griffiths-Jones S Saini HK Van Dongen S Enright AJ. Mirbase: tools for microrna genomics. Nucleic Acids Res. (2008) 36:D154–8. 10.1093/nar/gkm952 17991681 PMC2238936

[18] Friedlander MR Mackowiak SD Li N Chen W Rajewsky N. Mirdeep2 accurately identifies known and hundreds of novel microrna genes in seven animal clades. Nucleic Acids Res. (2012) 40:37–52. 10.1093/nar/gkr688 21911355 PMC3245920

[19] Anders S. Analysing RNA-Seq data with the Deseq package. Mol Biol. (2010) 43:1–17.25337456

[20] Enright AJ John B Gaul U Tuschl T Sander C Marks DS. Microrna targets in *Drosophila*. Genome Biol. (2003) 5:R1. 10.1186/gb-2003-5-1-r1 14709173 PMC395733

[21] Kong D Chen T Zheng X Yang T Zhang Y Shao J. Comparative profile of exosomal micrornas in postmenopausal women with various bone mineral densities by small RNA sequencing. Genomics. (2021) 113:1514–21. 10.1016/j.ygeno.2021.03.028 33785399

[22] Ashary N Tiwari A Modi D. Embryo implantation: war in times of love. Endocrinology. (2018) 159:1188–98. 10.1210/en.2017-03082 29319820

[23] Gaonac'h-Lovejoy V Boscher C Delisle C Gratton JP. Rap1 is involved in angiopoietin-1-induced cell-cell junction stabilization and endothelial cell sprouting. Cells. (2020) 9:155. 10.3390/cells9010155 31936361 PMC7016689

[24] Albayrak IG Azhari F Colak EN Balci BK Ulgen E Sezerman U. Endometrial gene expression profiling of recurrent implantation failure after *in vitro* fertilization. Mol Biol Rep. (2021) 48:5075–82. 10.1007/s11033-021-06502-x 34216338

[25] Luan L Ding T Stinnett A Reese J Paria BC. Adherens junction proteins in the hamster uterus: their contributions to the success of implantation. Biol Reprod. (2011) 85:996–1004. 10.1095/biolreprod.110.090126 21753191 PMC3197917

[26] Wang X Matsumoto H Zhao X Das SK Paria BC. Embryonic signals direct the formation of tight junctional permeability barrier in the decidualizing stroma during embryo implantation. J Cell Sci. (2004) 117:53–62. 10.1242/jcs.00826 14627626

[27] Zhang Y Yang Z Wu J. Signaling pathways and preimplantation development of mammalian embryos. FEBS J. (2007) 274:4349–59. 10.1111/j.1742-4658.2007.05980.x 17680809

[28] Salilew-Wondim D Gebremedhn S Hoelker M Tholen E Hailay T Tesfaye D. The role of micrornas in mammalian fertility: from gametogenesis to embryo implantation. Int J Mol Sci. (2020) 21:585. 10.3390/ijms21020585 31963271 PMC7014195

[29] Wu F Mao D Liu Y Chen X Xu H Li TC. Localization of Mucin 1 in endometrial luminal epithelium and its expression in women with reproductive failure during implantation window. J Mol Histol. (2019) 50:563–72. 10.1007/s10735-019-09848-6 31555952

[30] Poon CE Madawala RJ Dowland SN Murphy CR. Nectin-3 is increased in the cell junctions of the uterine epithelium at implantation. Reprod Sci. (2016) 23:1580–92. 10.1177/1933719116648216 27217376

[31] Su R-W Jia B Ni H Lei W Yue S-L Feng X-H. Junctional adhesion molecule 2 mediates the interaction between hatched blastocyst and luminal epithelium: induction by progesterone and Lif. PLoS ONE. (2012) 7:e34325. 10.1371/journal.pone.0034325 22511936 PMC3325240

[32] Ye X Herr DR Diao H Rivera R Chun J. Unique uterine localization and regulation may differentiate Lpa3 from other lysophospholipid receptors for its role in embryo implantation. Fert Ster. (2011) 95:2107–13. 10.1016/j.fertnstert.2011.02.024 21411082 PMC3084612

[33] Boettner B Van Aelst L. Control of cell adhesion dynamics by Rap1 signaling. Curr Opin Cell Biol. (2009) 21:684–93. 10.1016/j.ceb.2009.06.004 19615876 PMC2841981

[34] Li Q Teng Y Wang J Yu M Li Y Zheng H. Rap1 promotes proliferation and migration of vascular smooth muscle cell *via* the ERK pathway. Pathol Res Pract. (2018) 214:1045–50. 10.1016/j.prp.2018.04.007 29789158

[35] Kurian NK Modi D. Extracellular vesicle mediated embryo-endometrial cross talk during implantation and in pregnancy. J Assist Reprod Genet. (2019) 36:189–98. 10.1007/s10815-018-1343-x 30362057 PMC6420537

[36] Wang X Li Q Xie T Yuan M Sheng X Qi X. Exosomes from bovine endometrial epithelial cells ensure trophoblast cell development by MIR-218 targeting secreted frizzled related protein 2. J Cell Physiol. (2021) 236:4565–79. 10.1002/jcp.30180 33230823

[37] Kusama K Rashid MB Kowsar R Marey MA Talukder AK Nagaoka K. Day 7 embryos change the proteomics and exosomal micro-rnas content of bovine uterine fluid: involvement of innate immune functions. Front Genet. (2021) 12:676791. 10.3389/fgene.2021.676791 34262596 PMC8273763

[38] Nicola I. Mir-184 Has Multiple roles in drosophila female germline development. Dev Cell. (2009) 17:123–33. 10.1016/j.devcel.2009.06.008 19619497

[39] Song Y An X Zhang L Fu M Peng J Han P. Identification and profiling of micrornas in goat endometrium during embryo implantation. PLoS ONE. (2015) 10:e0122202. 10.1371/journal.pone.0122202 25886011 PMC4401794

[40] Cui J Liu X Yang L Che S Guo H Han J. Mir-184 combined with stc2 promotes endometrial epithelial cell apoptosis in dairy goats *via* Ras/Raf/Mek/Erk pathway. Genes. (2020) 11:1052. 10.3390/genes11091052 32906580 PMC7565287

